# A Novel Case of Tenosynovial Giant Cell Tumor at 4 Years After Total Knee Arthroplasty for Medial Osteoarthritis With Synovial Osteochondromatosis

**DOI:** 10.1155/cro/9220974

**Published:** 2025-05-09

**Authors:** Yu-uichi Mishima, Takao Kaneko, Kosuke Shiga, Ayakane Yamamoto, Shu Yoshizawa

**Affiliations:** Adult Reconstruction Center, Ichinomiya Onsen Hospital, Fuefuki, Yamanashi, Japan

**Keywords:** synovectomy, synovial osteochondromatosis, tenosynovial giant cell tumor, three-dimensional computed tomography, total knee arthroplasty

## Abstract

We report an unusual case of a tenosynovial giant cell tumor (TGCT) in a patient who received a total knee arthroplasty (TKA) for medial knee osteoarthritis with synovial osteochondromatosis (SO). A 72-year-old woman underwent conventional jig-based simultaneous bilateral bicruciate-stabilized TKA. Four years after the surgery, she came to our hospital with a chief complaint of persistent pain in the right knee only, with recurrent spontaneous hemarthrosis. We performed a synovectomy with open excisional debridement and a polyethylene exchange. Histologic analysis of synovial samples was consistent with TGCT. We present the imaging and pathological details of the first case of de novo TGCT that occurred in the background of SO after TKA.

## 1. Introduction

The presentation of tenosynovial giant cell tumor (TGCT) after total knee arthroplasty (TKA) has been previously described in the literature [1, 2]. There are currently no published reports describing at 4 years after TKA performed for knee osteoarthritis with concomitant synovial osteochondromatosis (SO).

SO is a rare, proliferative but benign disease of the synovial membrane of joints, tendon sheaths, or bursae that can cause severe dysfunction of the affected synovial joints [3]. SO is most common in men, typically presents during the third to fifth decade of their life, and primarily affects large joints such as the knee or hip [4]. Treatment of SO, which has a low recurrence rate, involves open or arthroscopic removal of loose bodies with complete or partial synovectomy. TGCT is a benign synovial joint proliferative disorder with an incidence of 1.8 million cases per year [5], and most cases occur in the synovial membrane of large joints such as the knee and hip [6]. Its etiology remains inconclusive, although recurrent hemorrhage, neoplasm, and trauma have been reported as possible causes [7]. There are two types of TGCT, namely localized and diffuse [8]. The diffuse-type TGCT is the new world health organization (WHO) terminology. The localized form is characterized by a discrete mass within the synovium, as opposed to diffuse TGCT, which involves the entire synovium and can be either intra-articular or extra-articular [9]. Both SO and TGCT are considered to be tumor-like diseases. Here, we experienced an unusual case of de novo TGCT that occurred in the background of SO after primary TKA for knee osteoarthritis.

## 2. Case Presentation

**Figure 1:**
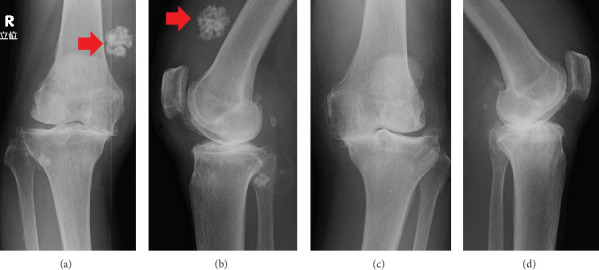
Preoperative plain radiograph with osteoarthritis of the bilateral knees. (a, b) Right knee and (c, d) left knee. (a, c) Anteroposterior view and (b, d) lateral view of the bilateral knees. Synovial osteochondromatosis was confirmed on the right knee (red arrow).

**Figure 2:**
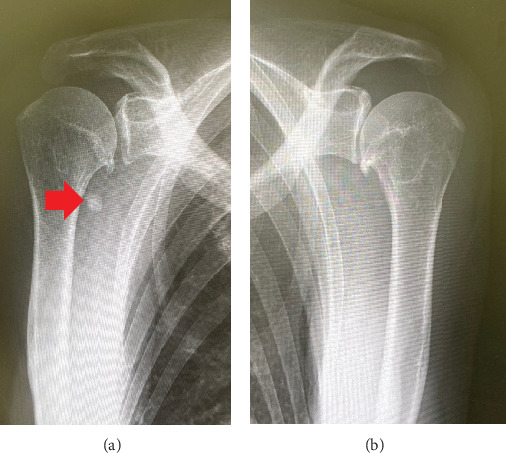
Plain radiographs of the bilateral shoulder joints. (a) Right knee and (b) left knee. (a, b) Anteroposterior view. Synovial osteochondromatosis was confirmed on the right side.

**Figure 3:**
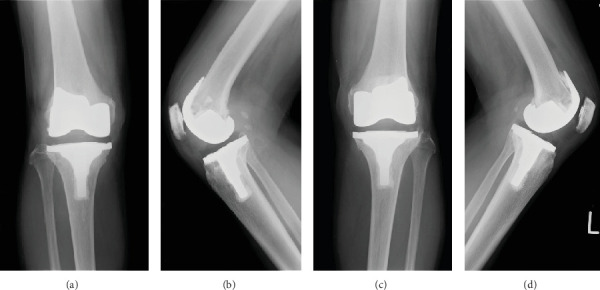
Postoperative plain radiograph after simultaneous bilateral bicruciate-stabilized total knee arthroplasty. (a, b) Right knee and (c, d) left knee. (a) Anteroposterior view, (b) lateral view of the right knee, (c) anteroposterior view, and (d) lateral view of the left knee.

A 72-year-old female farmer with a body mass index of 21.5 kg/m^2^ presented with knee swelling, pain, and limited range of motion. Radiographs showed significant osteoarthritis with marked medial joint space narrowing bilaterally and substantial SO with multiple round calcifications in the suprapatellar bursa on the right side (Figure 1). SO also involved the shoulder on the right side (Figure 2). We performed a conventional jig-based simultaneous bilateral TKA using a bicruciate-stabilized prosthesis (Journey II BCS, Smith & Nephew Inc., Memphis, Tennessee, United States) (Figure 3). Perioperatively, numerous corpora libra were extracted, and synovectomies were performed as far as possible.

**Figure 4:**
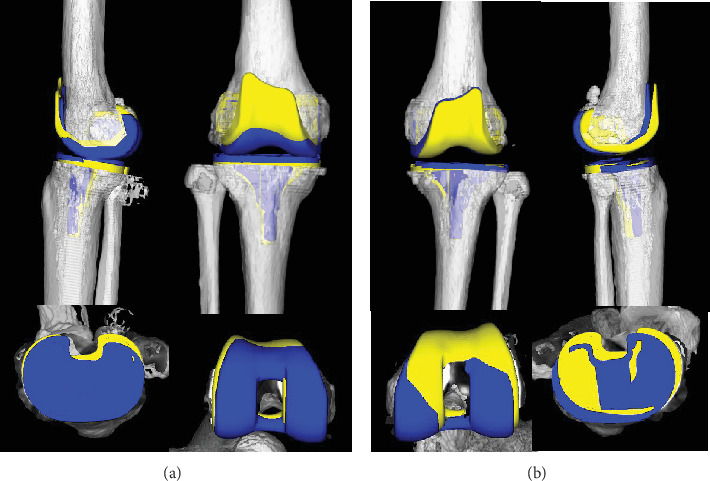
Preoperative three-dimensional computed tomography (3DCT) plan and postoperative 3DCT images were shown. 3D computer-aided design (CAD) data of femoral and tibial components were fitted to the 3DCT plan and images. Preoperative plan components are shown in blue. Postoperative components are shown in yellow. (a) Right knee and (b) left knee. The femoral component sagittal alignment in the left knee was 2° more flexed than the preoperative plan, but the right femoral component sagittal alignment in the right knee was similar to the preoperative plan.

Postoperative three-dimensional computed tomography (3DCT) images of the femur and tibia were superimposed onto those of the preoperative 3DCT plan targeting mechanically alignment using computer software (ZedView and ZedKnee; LEXI Co. Ltd., Tokyo, Japan) (Figure 4) [10–13]. The femoral component sagittal alignment in the left knee was 2° more flexed than the preoperative plan, but the right femoral component sagittal alignment in the right knee was similar to the preoperative plan. Patient-reported outcome measurements at 3 years after bilateral TKA were as follows for both knees: the 2011 Knee Society Score (symptoms: 25, patient satisfaction: 40, and daily activity: 100), the Western Ontario McMaster Universities Osteoarthritis Index score (pain: 20, stiffness: 8, and physical function: 68), the Forgotten Joint Score-12 (100), and the Patellar score (28).

**Figure 5:**
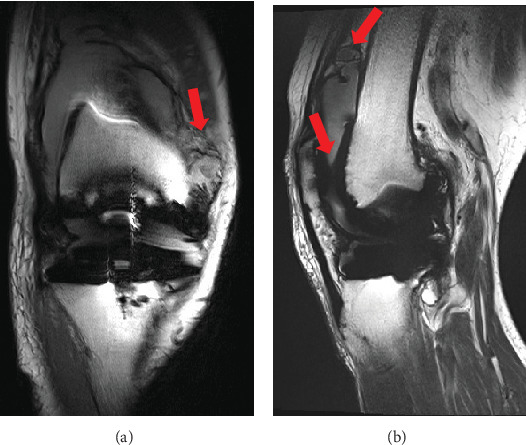
Magnetic resonance imaging in the right knee at 4 years after surgery. (a) Coronal view and (b) sagittal view of the right knee T2 low imaging demonstrating a moderate knee effusion (red arrow) and a low signal–high signal with hemorrhage in the suprapatellar and medial pouch (red arrow).

Four years postoperatively, the patient came to our hospital with a chief complaint of persistent pain in the right knee only, with recurrent spontaneous hemarthrosis. We aspirated 500 cc of synovial fluid repeatedly within a short period of time and found that it was bloody in appearance. Magnetic resonance imaging (MRI) of the right knee showed mixed high and low density associated with hemarthrosis and hemosiderin deposition (Figure 5).

**Figure 6:**
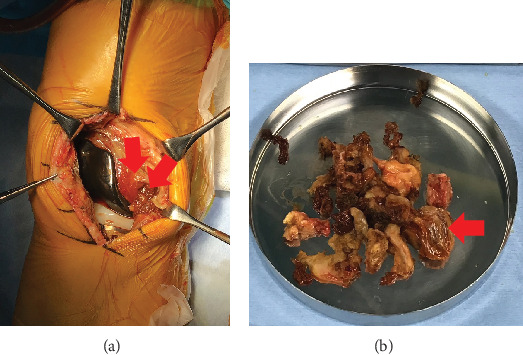
Intraoperative photograph in the right knee. (a) Knee in extension and (b) the removal of a large complex hematoma (red arrow). A black-brown hematoma was confirmed in the suprapatellar and medial pouch (red arrow).

**Figure 7:**
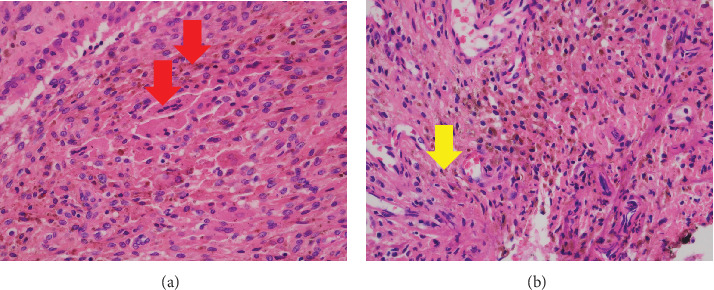
Pathological findings of the synovium analyzed intraoperatively. (a, b) enlarged images at 400× magnification. Proliferation in old hemosiderin deposits: histiocyte-like cells (red arrow) and mononuclear oval histiocytic cells and multinucleated giant cells (yellow arrow).

After removing the ultrahigh molecular weight polyethylene (UHMWPE), we performed an open synovectomy with a total excision of the hematoma which is associated with TGCT (Figure 6). No component aseptic loosening was found. There was no growth on operative cultures, and histologic analysis of synovial samples was consistent with TGCT by a soft tissue tumor specialist pathologist (Figure 7). We diagnosed SO with transformation into TGCT after TKA.

## 3. Discussion

We performed an open synovectomy for a rare case of de novo TGCT that occurred in the background of SO following primary TKA for knee osteoarthritis.

SO is typically monoarticular and usually affects the knee, but the hip, ankle, and elbow can also be involved [14]. Plain-film radiography and clinical findings are essential for its diagnosis. In this case, SO affected the knee and shoulder joint on the right side. In 1977, Milgram divided SO into the following three successive classification: Type 1: intrasynovial disease without loose bodies, Type 2: transitional disease with active intrasynovial proliferation and free loose bodies, and Type 3: multiple free loose bodies without active intrasynovial proliferation [4]. Untreated SO may result in secondary arthritis due to mechanical injury caused by loose bodies and disruption of nutrient delivery to the articular cartilage [15]. The present case was Type 2 in Milgram classification and complicated by secondary osteoarthritis of the right knee. Ackerman et al. [16] described four SO patients who received TKA with good results. On the contrary, in the present case, SO changed to PVNS rather than recurring in the right knee at 4 years after bilateral simultaneous TKA. TGCT is a locally aggressive proliferative disorder of the synovium that is a rare complication after TKA [17, 18]. Ballard et al. [18] first described TGCT that occurred in a patient 9 years after TKA. They hypothesized that UHMWPE wear along with the microtrauma of daily movement caused episodes of bleeding that resulted in pigmented synovitis [18]. In this case, we confirmed that there was no wear of the extracted UHMWPE. In addition, no malalignment was observed on postoperative 3DCT regarding UHMWPE wear due to impingement with the patellar component as the femoral component was in flexion.

Houdek et al. [19] reported a recurrence rate of 12% at 14-year follow-up among patients who had a previous diagnosis of TGCT before TKA; therefore, long-term follow-up is necessary.

To the best of our knowledge, this is the first case of de novo TGCT that occurred in the background of SO after TKA and contributes to the literature by reporting this rare occurrence.

We hypothesized that during TKA, synovectomy for SO due to chondrogenesis of the synovium induced a locally aggressive proliferative disorder of the synovium, resulting in TGCT. We need to encourage further reporting and discussion to fully understand the etiology of TGCT. The limitation of this case is that the initial TKA was performed based on radiographic rather than pathology to confirm the diagnosis of SO.

The clinical relevance of the present case is that the surgeon should keep TGCT in mind for a recurrent nontraumatic intra-articular hematoma after TKA for knee osteoarthritis with SO.

## Data Availability

The data that support the findings of this study are available from the corresponding author upon reasonable request.
